# Dual strain mechanisms in a lead-free morphotropic phase boundary ferroelectric

**DOI:** 10.1038/srep19630

**Published:** 2016-01-21

**Authors:** Julian Walker, Hugh Simons, Denis O. Alikin, Anton P. Turygin, Vladimir Y. Shur, Andrei L. Kholkin, Hana Ursic, Andreja Bencan, Barbara Malic, Valanoor Nagarajan, Tadej Rojac

**Affiliations:** 1 Electronic Ceramics Department, Jozef Stefan Institute, Ljubljana, Slovenia; 2 Department of Physics, Technical University of Denmark, Lyngby DK-2800 kgs. Denmark; 3 Nanofer Laboratory, Institute of Natural Sciences, Ural Federal University, Ekaterinburg, Russia; 4 CICECO & Department of Materials and Ceramics Engineering, University of Aveiro, Aveiro, Portugal; 5 The School of Materials Science and Engineering, University of New South Wales, Sydney, Australia

## Abstract

Electromechanical properties such as d_33_ and strain are significantly enhanced at morphotropic phase boundaries (MPBs) between two or more different crystal structures. Many actuators, sensors and MEMS devices are therefore systems with MPBs, usually between polar phases in lead (Pb)-based ferroelectric ceramics. In the search for Pb-free alternatives, systems with MPBs between polar and non-polar phases have recently been theorized as having great promise. While such an MPB was identified in rare-earth (RE) modified bismuth ferrite (BFO) thin films, synthesis challenges have prevented its realization in ceramics. Overcoming these, we demonstrate a comparable electromechanical response to Pb-based materials at the polar-to-non-polar MPB in Sm modified BFO. This arises from ‘dual’ strain mechanisms: ferroelectric/ferroelastic switching and a previously unreported electric-field induced transition of an anti-polar intermediate phase. We show that intermediate phases play an important role in the macroscopic strain response, and may have potential to enhance electromechanical properties at polar-to-non-polar MPBs.

The enhanced electromechanical response observed at (or near) a morphotropic phase boundary (MPB) is a critical phenomenon in ferroelectrics^1^,^2^. At an MPB, crystallographically different phases with low energy barriers coexist, realizing large physical responses (e.g., strain *ε* and polarization *P*) to weak external stimuli (e.g., stress *σ* and electric-field *E*)^3^,^4^. MPB materials are central to the booming market for piezoelectric devices, which is dominated by lead (Pb)-based materials such as Pb(Zr,Ti)O_3_ (PZT) and (1-x)PbMg_1/3_Nb_2/3_O_3_-(x)PbTiO_3_ (PMN-PT)^5^. Anti-Pb legislation and changing social attitudes towards environmental sustainability and toxic materials in waste and manufacturing, have instigated an intense global search for Pb-free ferroelectrics containing MPBs with large electromechanical responses^6^,^7^,^8^,^9^,^10^.

The exploration and design of MPBs between polar and non-polar phases (polar-to-non-polar MPB) is a fresh and radical approach to identifying and engineering Pb-free ferroelectrics that emulate both reliable synthesis and the electromechanical properties of PZT^11^. Although consistent with Goldschmidt´s definition of an MPB^12^, polar-to-non-polar MPBs are fundamentally different to the more widely recognized MPB between two polar phases (polar-to-polar MPB), such as between rhombohedral (*R*3*m*) and tetragonal (*P*4*mm*) (and intermediate monoclinic) phases in PZT^13^. At polar-to-polar MPBs, property enhancements are proposed to relate to the ease of polarization rotation under external electric fields^2^,^3^. At polar-to-non-polar MPBs however, such enhancement results from a polarization extension mechanism (associated with producing the largest piezoelectric response ever reported: d_16_ = 20 000 pm/V in KH_2_PO_4_)^11^. The presence of an intermediate polar phase was predicted to allow both polarization extension and rotation mechanisms to occur simultaneously and further enhance electromechanical performance. Polar-to-non-polar MPBs are therefore, a new class of materials which have tremendous potential as the new generation of high-performance Pb-free piezoelectrics.

A Pb-free, polar-to-non-polar MPB was recently demonstrated in rare-earth (RE)-modified bismuth ferrite (BiFeO_3_, BFO)^14^,^15^, where RE^3+^ ions (e.g. Sm, Nd, Gd, Dy) are isovalently substituted for Bi^3+^. BFO makes an exceptional candidate end-member for the polar-to-non-polar class of Pb-free MPBs due to its very high Curie transition temperature (*T*_*c*_ ~1100 K)^16^, high remanent polarization (*P*_*r*_ ~90 μC/cm^2^)^17^,^18^ and room-temperature multiferroicity^19^,^20^. Combinatorial thin-film studies revealed that RE-modified BFO (RE-BFO) exhibits enhanced piezoelectric coefficients (*d*_*33*_) and reduced coercive fields (*E*_*c*_) at an MPB between polar, rhombohedral (*R*3*c*) and non-polar, orthorhombic (*Pnma*) phases^14^,^15^. However, the success of RE-BFO thin-films has not been replicated by polycrystalline ceramics, despite their much greater demand in commercial applications such as sensors, actuators and transducers. Instead, their development has been slowed due to the processing problems and high electrical conductivity of BFO and related materials^21^. Realizing the potential of RE-BFO therefore requires robust electromechanical properties in polycrystalline ceramics.

The aim of this work is to demonstrate just this, and to identify the structural and morphological conditions accompanying polar-to-non-polar MPBs. Here, in polycrystalline materials of the prototypical RE-BFO system Bi_1-x_Sm_x_FeO_3_ (BSFO), we achieve switching strains of ~0.3% (peak-to-peak (*S*_*pp*_) at electric-field frequency of 100 Hz) comparable to those of established Pb-based systems^22^,^23^,^24^,^25^. Through a systematic study across the MPB, with compositions x = 8–18 mol% Sm, we show how an anti-polar phase^26^,^27^ underpins the evolution of the phase composition and crucially, drives a nano-scale domain topology with increasing Sm content towards the MPB (nearest studied composition 15.5 mol% Sm). A strain-electric-field study reveals that the anti-polar phase facilitates dual strain mechanisms; both ferroelectric/ferroelastic domain switching and a previously unreported electric-field induced phase transition. This transition of an anti-polar intermediate phase is the key to understanding their role in RE-BFO and discloses an important opportunity to achieve large electromechanical properties at polar-to-non-polar MPBs. By using polycrystalline materials we bring new understanding to RE-BFO, complementing the existing picture of atomic-scale evolution in thin films^28^, and in doing so we highlight the potential of polar-to-non-polar MPB systems as Pb-free piezoelectrics.

## Results

### Evolution of BSFO phase composition

**Figure 1:**
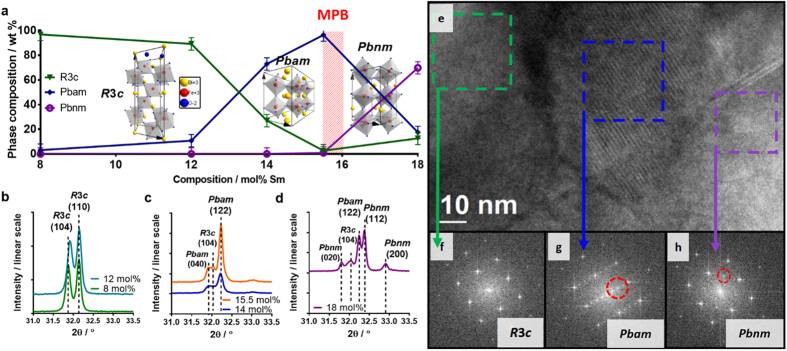
Crystal structural evolution. (**a**) Quantified phase composition (wt% of *R*3*c*, *Pbam* and *Pbnm* phases) as a function of the Sm composition (mol% Sm). Error bars indicate approximate XRD measurement error, the location of the polar-to-non-polar MPB is marked with red and the images of the unit cells corresponding to the space group of each phase present are shown. (**b**–**d**) Show the 2θ region 31–33.5° for 8–12 mol% Sm, 14–15 mol% Sm and 18 mol% Sm respectively, where the pseudo cubic (110)_pc_ peak occurs. The peaks are labelled according to their space group and corresponding peak indices. (**e**) High resolution (HR) transmission electron microscopy (TEM) image of the 15.5 mol% Sm sample with closest proximity to the PB region. (**f**–**h**) Show selected area fast Fourier transforms (FFTs) in [001]_pc_ zone axis of specific phase regions corresponding respectively to *R*3*c*, *Pbam* with ¼ (100)_pc_ reflections (ringed), and *Pbnm* with ½ (110)_pc_ reflections (ringed). These regions are marked in the HR-TEM image by dashed boxes.

The phase composition of BSFO ceramics evolves as a function of the Sm content (where each composition is defined by its molar percentage of Sm i.e., x mol% Sm) and is well documented in the literature^15^,^26^,^27^,^28^,^29^,^30^. Powder X-ray diffraction (XRD) is used to identify three phases with different crystal structures across the compositional range covered by the five BSFO compositions: 8, 12, 14, 15.5 and 18 mol% Sm (Fig. 1):

1. Phase 1: Polar, rhombohedrally distorted perovskite with space group *R*3*c*^16^. This phase is found predominantly in 8 and 12 mol% Sm (Fig. 1a,b).
2. Phase 2: Intermediate, orthorhombically distorted perovskite with an anti-polar A-site cation ordering and space group *Pbam*, isostructural with anti-polar PbZrO_3_^26^,^27^. *Pbam* coexists with the *R*3*c* phase and is most prevalent when the compositions have close proximity to the polar-to-non-polar MPB (Fig. 1a), i.e., 14 and 15.5 mol% Sm (Fig. 1a,c).
3. Phase 3: Non-polar, orthorhombic structure with space groups *Pnma*, or *Pbnm* (here considered equivalent), isostructural with SmFeO_3_ and Sm-rich (Bi_1-x_Sm_x_)FeO_3_^31^. This is the phase appearing in the non-polar region of the phase diagram^26^,^27^ found in compositions after the polar-to-non-polar MPB, i.e., 18 mol% Sm (Fig. 1a and d). Due to its location in the non-polar region of the phase diagram, the 18 mol% Sm composition is not discussed further.

(For further details of structural parameters see supplementary materials A).

Firstly, 8, 12, 14 and 15.5 mol% Sm compositions are within a region where *R*3*c* (Phase 1) and *Pbam* (Phase 2) coexist, and the ratio of *Pbam* to *R*3*c* phase increases with the Sm content (Fig. 1a–c). Previous transmission electron microscopy (TEM) studies have revealed that this *R*3*c–Pbam* phase coexistence occurs on a nanoscale level, with individual chemically homogeneous grains consisting of an intimate nanoscale *R*3*c–Pbam* phase mixture^32^. The 15.5 mol% Sm composition has the closest compositional proximity to the polar-to-non-polar MPB, which exists between the region of coexisting *R*3*c*–*Pbam* (Phase 1–Phase 2), and the non-polar *Pbnm* (Phase 3). The nanoscale coexistence between *R*3*c* and *Pbam* phases in compositions close to the polar-to-non-polar MPB is believed to be a prerequisite for the enhanced electromechanical behavior observed in RE-BFO^29^.

In the composition closest to the MPB (15.5 mol% Sm), all three phases (*R*3*c*, *Pbam* and *Pbnm*) are present on the nano-scale (Fig. 1e–h). We note that despite no chemical inhomogeneity being detected^32^, we cannot rule out the contribution of both equilibrium and non-equilibrium effects in producing the phase coexistence observed. Nonetheless, the similarity between the phase coexistence of the BSFO ceramics and epitaxial thin films^30^, despite radically different synthesis conditions, is clear evidence for the effectiveness of the mechanochemical activation synthesis method used here (discussed in ref. 32) and additionally demonstrates the value of combinatorial thin-film studies as a rapid structural prototyping tool.

**Figure 2:**
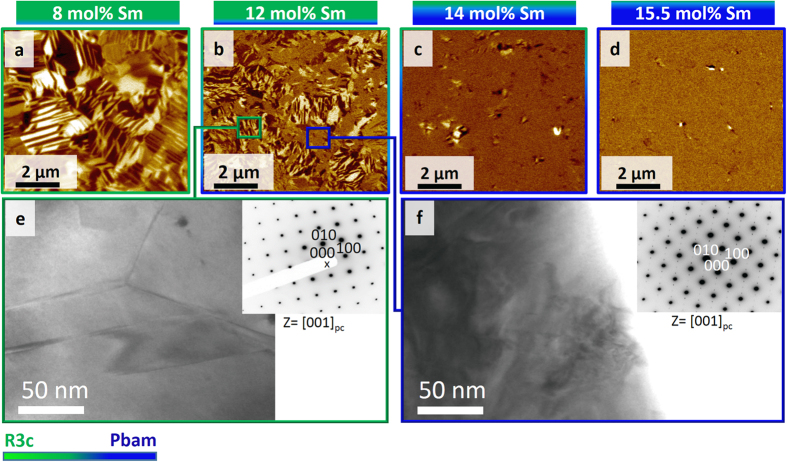
Evolution of domain topology. (**a**–**d**) Piezoresponse force microscopy (PFM) out-of-plane amplitude images obtained for 8 mol% Sm, 12 mol% Sm, 14 mol% Sm and 15.5 mol% Sm respectively. (**e**,**f**) Show Bright field (BF) – TEM images and associated selected area electron diffraction (SAED) patterns from 12 mol% Sm. (**e**) Shows a region identified as *R*3*c* by corresponding SAED in [001]_pc_ zone axis (inset), where regular domains are seen. (**f**) Shows a region identified as *Pbam* phase by corresponding SAED (inset), where complicated nano-sized features are observed. The green-to-blue color transition approximately represents the change in wt% ratio of *R*3*c* and *Pbam* phase. PC denotes pseudocubic notation.

The change of the ferroelectric domain structure as a function of the increasing Sm content, is imaged by piezoresponse force microscopy (PFM) (Fig. 2a–d) and, reflects the evolution of the phase composition (Fig. 1a). At 8 mol% Sm, the domain topology is mainly comprised of regular lamella and wedges of size ~100–500 nm, reminiscent of those observed in unmodified BFO^33^. As the Sm content is increased for 12 and 14 mol% Sm, the domains become progressively smaller (~50–200 nm) and their topology more irregular. The number of visible domains is also reduced as the composition approaches the MPB (from 8–14 mol% Sm), to the extent that almost no domains are visible at 15.5 mol% Sm (Fig. 2d). We note that the grain size remains consistent across all compositions (~1–3 μm) (further details in supplementary materials B).

Bright-field (BF)-TEM and selected area electron diffraction (SAED) directly relate the reduction in size and visibility of the domains to the emergence of the *Pbam* phase as the Sm content is increased (Fig. 2e,f). Phase regions identified by SAED with *R*3*c* structure exhibit clear lamella domains (Fig. 2e). Further confirmation of this domain structure is given in supplementary materials B. Phase regions with *Pbam* structure, exhibit irregular features with a size scale of several tens of nanometers (Fig. 2f), meaning they are beneath the size detection limits of PFM^34^. The anti-polar *Pbam* phase is also centrosymmetric (with point group *mmm*) and is not expected to exhibit a PFM response. Finally, the *Pbam* phase forms as nano-sized clusters within *R*3*c* grains^32^,^35^ disrupting the domain structure and resulting in smaller *R*3*c* domains. The increasing *Pbam* phase content (Fig. 1a) thus explains the reducing size and number of domains as the Sm content is increased (Fig. 2a–d).

### Electric-field induced phase transition

**Figure 3:**
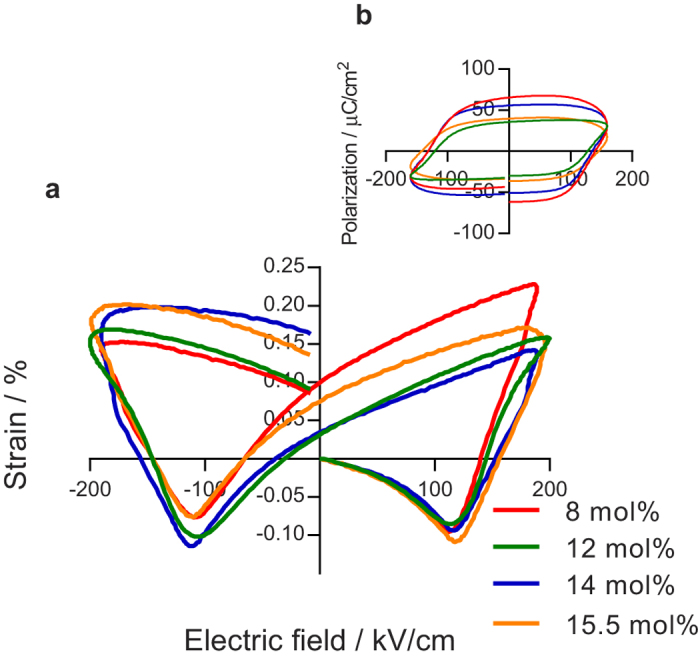
Hysteresis behavior of different BSFO compositions. (**a**) S-E hysteresis for 8, 12, 14, and 15.5 mol% Sm measured at 200 kV/cm and 100 Hz. (**b**) shows polarization-electric-field (**P**–**E**) loops with maximum field amplitude of 160 kV/cm and 100 Hz.

For BSFO compositions 8–15.5 mol% Sm, we observe a similar ˝butterfly˝-like shape of the strain-electric-field (S-E) hysteresis with consistent strains (*S*_*pp*_ of ~0.3 ± 0.03%) and coercive field values (*E*_*c*_ of ~130 kV/cm) (Fig. 3). The S-E responses shown in Fig. 3a were recorded after many (10–20) bipolar electric-field cycles and represent an approximately saturated switched state (see method). This behavior is analogous with other ferroelectrics with strains arising from ferroelectric/ferroelastic domain switching^5^,^22^,^36^. The corresponding polarization-electric-field (P-E) responses (Fig. 3b) include contributions from electrical leakage currents characteristic of materials with a high BFO content^21^,^29^. Nonetheless, we emphasize that the *S*_*pp*_ recorded for the BSFO piezoceramics (~0.3%) are comparable to PZT^22^,^23^ and PMN-PT^24^,^25^, which in itself is a promising achievement in the development of Pb-free piezoceramics. We also note that BSFO ceramics exhibit comparable strain magnitudes to unmodified BFO ceramics (0.36%), when BFO is driven by a low electric-field frequency (i.e. 0.1 Hz)^37^. However, BFO strain behavior exhibits a strong frequency dependence, presumably related to strong domain-wall pinning effects^38^, that results in a significantly reduced strain of <0.1% at frequencies of 100 Hz, which is less than half that of BSFO ceramics (~0.3%).

**Figure 4:**
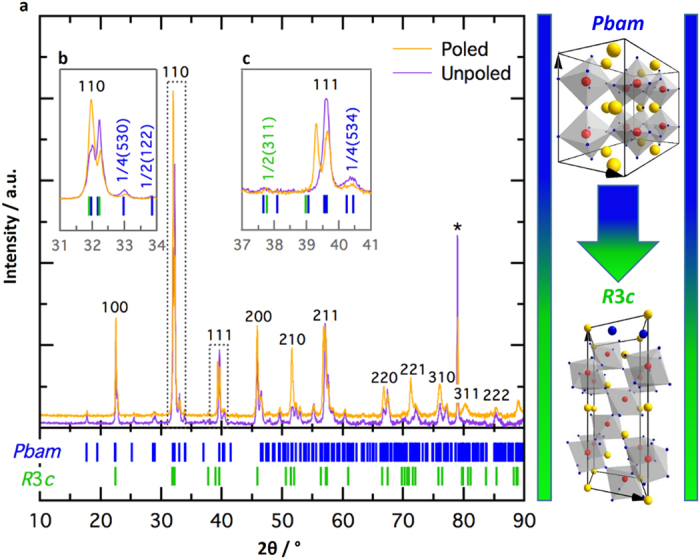
*Pbam*-to-*R*3*c* electric-field induced phase transition in 15.5 mol% Sm. (**a**) XRD pattern in the 2 theta range 10–90°. Peaks are index according to pseudocubic notation. (**b**) Relative intensity change of (110)_pc_ reflection and its neighboring satellites, signifying a reduction in the *Pbam* phase fraction. (**c**) Splitting of the (111)_pc_ peak upon poling, indicating induced long-range rhombohedral order. *Marks the peak arising from the sample holder during measurement. On the right are the unit cell structures of *Pbam* and *R*3*c* (drawn with Diamond®) and a blue-to-green color transition representing the change in phase ratio resulting from the phase transition.

**Figure 5:**
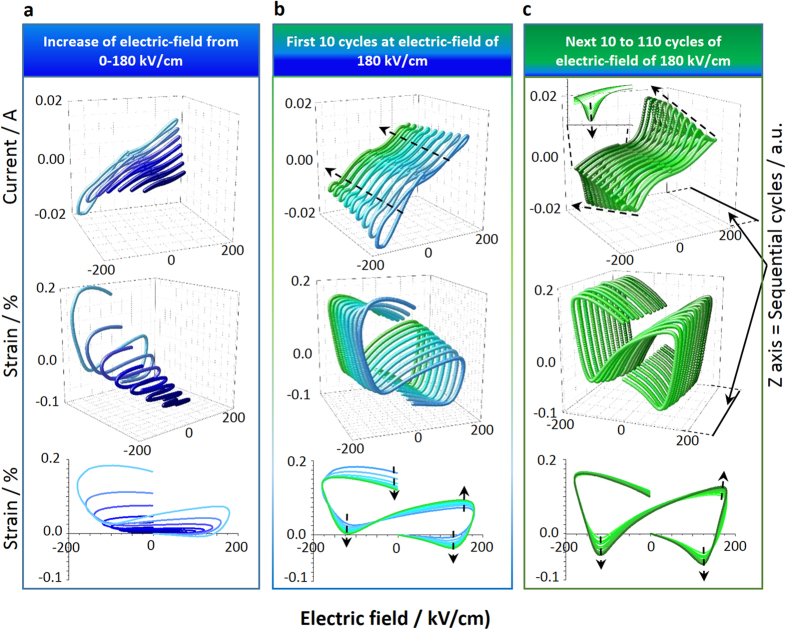
S-E hysteresis behavior of 15.5 mol% Sm as a function consecutive cycles of an electric-field with 100 Hz of frequency. The first two rows of graphs show I-E and S-E loops in 3-dimensional (3-D) plots, where the z axis corresponds to of number subsequent cycles in numerical order. The third, (bottom) row of graphs show S-E hysteresis in 2-D plots where the loop colors correspond to identical loops in 3-D plots. Arrows on I-E loops indicate increasing current peaks and arrows on S-E loops indicate the direction of S-E loop evolution with successive cycling (**a**) Loops observed during incrementally increasing the electric-field from 0–180 kV/cm with 10 kV/cm increments. (**b**) Loops observed during first 10 cycles of electric-field at 180 kV/cm. (**c**) Every 10^th^ loop observed (i.e. 20^th^, 30^th^, etc.) during sequential electric-field cycling from the 20^th^–110^th^ cycles.

In contrast to the domain topology (Fig. 2), the electromechanical behaviors (*S*_*pp*_ and saturated S-E responses in Fig. 3a) do not evolve significantly as a function of the Sm content, despite anti-polar *Pbam* emerging as the dominant phase (Fig. 1). This is unusual, as one might expect the emergence of an anti-polar phase to significantly influence macroscopic strain behavior^39^. However, this is explained by an electric-field induced phase transition of the *Pbam* phase to the *R*3*c* phase which, until now has been unreported in RE-BFO. We first discuss this phase transition under the application of a DC electric-field (Fig. 4), before showing how it manifests in the S-E behavior during the application of bipolar electric-fields (Fig. 5). This then provides a comprehensive picture of the role of this transition in the macroscopic strain behavior.

The *Pbam*-to-*R*3*c* phase transition is revealed by comparing XRD patterns of the near MPB composition (15.5 mol% Sm) before and after poling with a 120 kV/cm DC electric-field (Fig. 4a–c). The permanent macroscopic polarization of the material after poling was confirmed by direct measurement of d_33_ (~25 pC/N). The *d*_*33*_ achieved was intentionally lower than the maximum observed for this composition (*d*_*33*_^max^ ~50 pC/N), in order not to risk undermining the sample integrity during poling. There are two critical indicators of the electric-field induced phase transition from *Pbam*-to-*R*3*c* phase in the XRD patterns: a) the reduced intensity of the ¼(530)_pc_ reflection (where ‘pc’ denotes pseudo-cubic indexing), which is a satellite peak unique to the *Pbam* phase (Fig. 4b), and b) the splitting of the (111)_pc_ reflection, which corresponds to a long range distortion in the <111>_pc_ direction and is not allowable by *Pbam* symmetry (Fig. 4c).

### *Dual* strain mechanisms

We now establish how the electric-field induced *Pbam*-to-*R*3*c* phase transition and ferroelectric/ferroelastic domain switching of the R3c phase, constitute dual strain mechanisms in near MPB compositions (i.e., 15.5 mol% Sm) during repeated bipolar electric-field cycling from the virgin state (no prior electrical history). This is primarily evidenced by the evolution of current-electric-field (I–E) and S-E responses (Fig. 5 and supplementary materials C). Figure 5 is organized into three sections showing the evolution of I-E and S-E hysteresis loops during the initial increase of electric-field amplitude (Fig. 5a), and subsequent repeated cycling at electric-fields with amplitudes of 180 kV/cm (Fig. 5b,c).

As the electric-field magnitude is increased by 10 kV/cm increments in the range from 0–180 kV/cm (Fig. 5a), both the S-E and I-E loops show features that are inconsistent with typical ferroelectric/ferroelastic switching behavior^40^. The S-E loops possess an ˝S˝-like shape which becomes larger with increase of the electric-field amplitude and indicates expansion of the sample parallel to the applied electric-field, irrespective of the electric-field polarity. Small negative strains (sample contraction) are also evident in the low electric-field region of the S-E loops once the electric-field amplitude has increased beyond 120 kV/cm. This coincides with the E_c_ at ~130 kV/cm (determined from Fig. 3a) which suggests that the small negative strains are associated with the piezoelectric response of poled regions of *R*3*c* phase. The overall result is a strain response with a remanent strain as high as ~0.18% after the first cycle at 180 kV/cm. The corresponding I-E loops do not display clear current peaks characteristic of ferroelectric domain switching^41^, which strongly suggest that the positive strain observed in Fig. 5a arises from the *Pbam*-to-*R3c* phase transition. The electric-field induced phase transition is thus the first of the dual strain mechanisms to occur and does so in a lower electric-field range than the ferroelectric/ferroelastic domain switching, which has an average *E*_*c*_ of ~130kV/cm (see Fig. 3).

Electric-field induced phase transitions accompanied by large volume changes are an interesting alternative mechanism for strain, already observed with some anti-ferroelectric materials^39^, (Pb(Zr,Sn,Ti)O_3_^5^ and (Pb,Ba)(Zr,Nd,Ti)O_3_^42^), Pb-free materials ((Bi,Na)TiO_3_-BaTiO_3_-(KNa)NbO_3_^43^), and at epitaxial strain-induced MPBs in BFO^44^,^45^. The mechanisms involved with these transitions can be complex and facilitate transitions between many space group configurations^39^,^46^, particularly in BFO where pressure can also induce a number of phase transitions^47^. Here, the electric-field induced phase transition from *Pbam*-to-*R*3*c* phase involves a maximum unit cell strain of ~0.8% in the <111>_pc_ direction, determined from a simple calculation of the relative difference in the {111}_pc_ d spacing of the *Pbam* (~2.27 Å) and *R*3*c* (~2.29 Å) phases in the XRD pattern of the poled material (calculation in supplementary materials D).

Ferroelectric/ferroelastic domain switching emerges as a second source of strain after repeated electric-field cycling, at high electric-field magnitudes (180 kV/cm) in the 15.5 mol% Sm composition (Fig. 5b). In the first 10 electric-field cycles the S-E loop shapes evolve progressively and become more ˝butterfly˝-like with each subsequent cycle. The regions of the S-E loop with a negative slope (indicating contraction of the sample parallel to the direction of the applied electric-field) become more pronounced and the remanent strain reduces with further electric-field cycling. In the accompanying I-E loops, small peaks attributable to ferroelectric switching currents emerge and increase in intensity with each subsequent cycle (indicated by arrows in I-E curves in Fig. 5b).

Ferroelectric/ferroelastic domain switching becomes the dominant mechanism for strain in 15.5 mol% Sm with further electric-field cycling from 10–110 cycles (Fig. 5c). As the distinctive ˝butterfly˝-like shape of the S-E loop continues to develop, the peak intensities of the I-E loops also continue to increase significantly (indicated by arrows in I-E curves in Fig. 5c). The rate of development of both the S-E and I-E loops decreases with further electric-field cycling.

The S-E and I-E loops capture the shifting dominance of the dual strain mechanisms from electric-field induced phase transition to ferroelectric/ferroelastic domain switching. The ˝S˝-like shape of the initial S-E loops (Fig. 5a) is likely a manifestation of the strain contribution from the electric-field induced phase transition. While the ˝butterfly˝-like shape, which develops with further electric-field cycling (Fig. 5b,c), is characteristic of the switching of the ferroelectric/ferroelastic domains in the *R*3*c* phase.

## Discussion

Facilitated by the recent development of reliable synthesis methods, we demonstrate the first significant electromechanical strain responses in polycrystalline compositions of RE-BFO near to a polar-to-non-polar MPB. We discover a previously overlooked electric-field induced phase transition between *Pbam* and *R3c* phases in a composition with close proximity to the MPB and demonstrate the apparently significant contribution of the phase transition to the macroscopic strain behavior. We show how the unique coexistence of *R*3*c* and *Pbam* phases close to the polar-to-non-polar MPB determines the cycle-dependent strain evolution, which possesses the dual strain mechanisms of electric-field induced phase transition and ferroelectric/ferroelastic domain switching. This work therefore divulges the previously unresolved role of the *Pbam* phase in RE-BFO polar-to-non-polar MPB compositions.

Although the strain observed (S_pp_ ~0.3%) is already comparable to that of commercialized piezoelectrics, it is foreseeable that by maximizing the contributions of both dual mechanisms a pathway to large strain responses in this Pb-free material may be possible, drawing from a maximum unit cell distortion of ~0.8%. We hypothesize that the key approach for maximizing the electric-field induced strain response lies in the navigation of the complex intergranular elastic strains in polycrystalline materials. Thus, engineering domain structures, directionally orientating grains, utilizing flexoelectric effects or producing single crystal materials may sufficiently alleviate strain constrictions and facilitate the production of functional large strains.

## Methods

The ceramics were prepared by a mechanochemical-activation-assisted technique, as described by Walker *et al.*^32^. After mechanochemical activation the powder mixtures were uniaxially pressed and reactively sintered at 800 °C for 4 hours. Bulk densities were in the range of 7970–8190 kg/m^3^ (~95% of theoretical density) and average grain sizes in the range ~1–3 μm.

XRD patterns were recorded using a Bruker D8 Discover diffractometer in 10–90° 2θ range, a step of 0.04° and an acquisition speed of ~1.5°/min. Structure quantification was performed via whole-pattern Rietveld refinement using TOPAZ R software package (Version 2.1, 2003, Coelho software). For structural fitting three phases were used: (i) *R*3*c* phase (ICSD#15299), (ii) *Pbam* phase (ICSD #160460) and (iii) *Pbnm* phase (ICSD#162895). Structural evolution analysis was conducted on crushed pellets. The *ex-situ* electric-field structural study was conducted on sintered pellets with the electrodes removed before XRD and with acquisition speed of ~0.5°/min.

TEM was performed with JEOL JEM 2010 equipped with a JEOL EDXS detector. The specimens were prepared by mechanical grinding, dimpling and final Ar-ion milling. The piezoresponse images were recorded with an atomic force microscope (AFM; Asylum Research, Molecular Force Probe 3D, Santa Barbara, CA, USA) equipped with a piezoresponse force mode (PFM). A tetrahedral Si tip coated with Ti/Ir was used with a radius of curvature ~20 nm on a Si cantilever coated with Ti/Ir (Asyelec, AtomicForce F&E GmbH, Mannheim, Germany) and DPE-16 with Pt coating (DPE-16, Mikromasch, Estonia). AC voltage in range of 5–20 V was applied to AFM tip. Both the Dual AC Resonance Tracking Switching Spectroscopy (DART-SS) mode and an out-of-resonance regime with frequency ~20 kHz were used.

Ferroelectric switching and electromechanical properties were measured on samples ~0.2 mm thick with sputtered coated gold electrodes. An *axiACCT* TF 2000 analyzer with a *SIOS* meβtechnik laser interferometer and a *TREK* model 609E-6 (4 kV) high-voltage amplifier were used for measurements. Electric-fields were applied in a sinusoidal wave form. S-E loops measured at 200 kV/cm were generated with ˝saturated˝ switched materials, where ˝saturated˝ refers to the fact that samples were cycled at high electric-fields (~200 kV/cm) until only minimal increases of the *S*_*pp*_ were observed with each successive cycle (usually 10–20 cycles). DC poling was conducted with a *Spellman* SL150 amplifier, at room temperature in a silicone oil bath. *d*_*33*_ was determined with a *Take Control*® piezometer system PM10 at ~200 Hz driving stress frequency.

## Additional Information

**How to cite this article**: Walker, J. *et al.* Dual strain mechanisms in a lead-free morphotropic phase boundary ferroelectric. *Sci. Rep.* **6**, 19630; doi: 10.1038/srep19630 (2016).

## Supplementary Material

Supplementary Information
