# Supplementary material for: Dual strain mechanisms in a lead-free morphotropic phase boundary ferroelectric
Source: Sci Rep. 2016 Jan 21;6:19630. doi: 10.1038/srep19630 (PMC4726143; doi:10.1038/srep19630)
Supplement: Supplementary Information [file srep19630-s1.pdf]

Supplementary material

Dual strain mechanisms in a lead-free morphotropic phase boundary ferroelectric

Julian Walker[1,5,*], Hugh Simons[2], Denis O. Alikin[3], Anton P. Turygin[3], Vladimir Y. Shur[3], Andrei L. Kholkin[3,4], Hana Ursic[1], Andreja Bencan[1], Barbara Malic[1], Valanoor Nagarajan[5,+] & Tadej Rojac[1,+,*]

[1] Electronic Ceramics Department, Jozef Stefan Institute, Ljubljana, Slovenia

[2] Department of Physics, Technical University of Denmark, Lyngby DK-2800 kgs. Denmark

[3] Nanofer Laboratory, Institute of Natural Sciences, Ural Federal University, Ekaterinburg, Russia

[4] CICECO & Department of Materials and Ceramics Engineering, University of Aveiro, Aveiro, Portugal

[5] The School of Materials Science and Engineering, University of New South Wales, Sydney, Australia

[+] These authors contributed equally to this work

* Contact Authors: julian.walker@ijs.si (Julian Walker), Tadej.rojac@ijs.si (Tadej Rojac)

Supplementary material A

Crystal structure analysis: Rietveld refinement of X-ray diffraction (XRD) patterns

Supplementary Table A1. Structural parameters, phase compositions, goodness of fitting parameters of $(Bi_{1-x}Sm_x)FeO_3$ compositions determined by Rietveld refinement.

Composition (mol% Sm)	Identified Phase(s)	Phase composition (wt%)	Unit cell dimensions (Å)	Atom	*x*	*y*	*z*	R factors and GOF⁺
**8**	R3c	~95*	a=5.569	Bi	0.0000	0.0000	0.1078	GOF=1.58
			c=13.808	Sm	0.0000	0.0000	0.2496	R_{exp}=6.52
				Fe	0.0000	0.0000	0.3298	R_{wp}=10.30
				O	0.4430	0.0120	0.0654	R_p=7.43
**12**	R3c	87.7	a=5.569	Bi	0.0000	0.0006	0.0329	GOF=1.96
			c=13.788	Sm	0.0000	0.0024	0.0172	R_{exp}=5.71
				Fe	0.0000	0.0018	0.2572	R_{wp}=11.16
				O	0.0000	0.0086	0.0009	R_p=8.34
	Pbam	12.4	a=5.611	Bi	0.7183	0.1300	0.0000	
			b=11.203	Sm	0.7060	0.1236	0.5000	
			c=7.812	Fe	0.2396	0.0912	0.2336	
				O	0.2933	0.1878	0.0000	
				O	0.3886	0.0254	0.3719	
				O	0.0380	0.2260	0.2345	
				O	1.0000	0.5000	0.2435	
				O	0.0000	0.0000	0.2292	
**14**	R3c	27.1	a=5.556	Bi	0.0000	0.0000	0.0000	GOF=1.31
			c=13.751	Sm	0.0000	0.0000	0.0000	Rexp=7.46
				Fe	0.0000	0.0000	0.2212	Rwp=9.79
				O	0.4430	0.0120	0.9543	Rp=7.24
	Pbam	73.0	a=5.589	Bi	0.6968	0.1261	0.0000	
			b=11.213	Sm	0.7171	0.1257	0.5000	
			c=7.796	Fe	0.2372	0.1261	0.2549	
				O	0.2478	0.1757	0.0000	
				O	0.2805	0.8771	0.5000	
				O	0.0915	0.2726	0.3379	
				O	0.9534	0.5171	0.3169	
				O	0.0000	0.0000	0.2643	
**15.5**	Pbam	~95*	a=5.585	Bi	0.7183	0.1300	0.0000	GOF=2.01
			b=11.208	Sm	0.7060	0.1236	0.4999	R_{exp}=5.59
			c=7.795	Fe	0.2396	0.0911	0.2514	R_{wp}=11.22
				O	0.2933	0.1878	0.0000	R_p=7.80
				O	0.3886	0.0254	0.4388	
				O	0.0380	0.2260	0.2963	
				O	1.0000	0.5000	0.2038	
				O	0.0000	0.0000	0.2291	

**Composition (mol% Sm)**	**Identified Phase(s)**	**Phase composition (wt%)**	**Unit cell dimensions (Å)**	**Atom**	***x***	***y***	***z***	**R factors and GOF[+]**
**18**	Pbnm	69.9	a=5.446	Bi	0.9952	0.0461	0.2501	GOF=1.67
			b=5.622	Sm	0.9955	0.0465	0.2440	R_{exp}=6.07
			c=7.824	Fe	0.9745	0.5027	0.0158	R_{wp}=10.14
				O	0.1171	0.4617	0.2144	R_p=7.03
				O	0.7419	0.2410	0.0238	
	Pbam	17.6	a=5.583	Bi	0.7182	0.1300	0.0000	
			b=11.200	Sm	0.7060	0.1236	0.5000	
			c=7.799	Fe	0.2396	0.0912	0.2547	
				O	0.2933	0.1878	0.0000	
				O	0.3886	0.0254	0.5000	
				O	0.0380	0.2260	0.3379	
				O	1.0000	0.5000	0.3170	
				O	0.0000	0.0000	0.2643	
	R3c	12.6	a=5.554	Bi	0.0000	0.9910	0.01193	
			c=13.721	Sm	0.0000	0.0159	0.8701	
				Fe	0.0000	0.0003	0.2562	
				O	0.4430	0.0040	0.9771	

*Note: Where a single phase made up 95% or greater of the total phase wt% only the structural parameters of the majority phase have been included.

[+]GOF=Goodness of fit

Supplementary material B

Domain configurations in the R3c phase

The domain structure of $Bi_{1-x}Sm_xFeO_3$ (BSFO) compositions, where x = 8, 12, 14 and 15.5, is presented in the manuscript using both piezoresponse force microscopy (PFM) and transmission electron microscopy (TEM) (manuscript, **Fig. 2**). There is a correlation between the reducing domain size and the reducing number of domains as observed with PFM (manuscript, **Fig. 2a–d**), and the increasing content of *Pbam* phase in the crystal structure (observed with XRD, manuscript, **Fig. 1a**). The accompanying TEM and scanning area electron diffraction (SAED) data presented for 12 mol% Sm (manuscript, **Fig. 2e,f**) confirms, that regions with an R3c structure exhibit a regular domain structure, while regions with a *Pbam* structure exhibit nano-scale irregular features. The domain structure of regions of *R*3*c* phase in 15.5 mol% Sm ceramics is further confirmed by SAED patterns with zone axis $[001]_{pc}$ (**Supplementary Fig. B1**) (where pc symbolizes pseudo cubic notation). These SAED patterns exhibit (2 -3 0) reflection splitting in the $[100]_{pc}$ direction, indicative of 71° domains (**Supplementary Fig. B1 b**). The characteristic splitting was confirmed by simulations (**Supplementary Fig. B1 c**), which require domains in both the $[100]_{pc}$ and $[010]_{pc}$ zone axis in order to replicate the reflection splitting observed experimentally. A similar technique was used to identify the domain orientations present in unmodified $BiFeO_3$ (BFO) ceramics (Rojac et al., *J. Appl. Phys.* **112**, 064114-1, 2012).

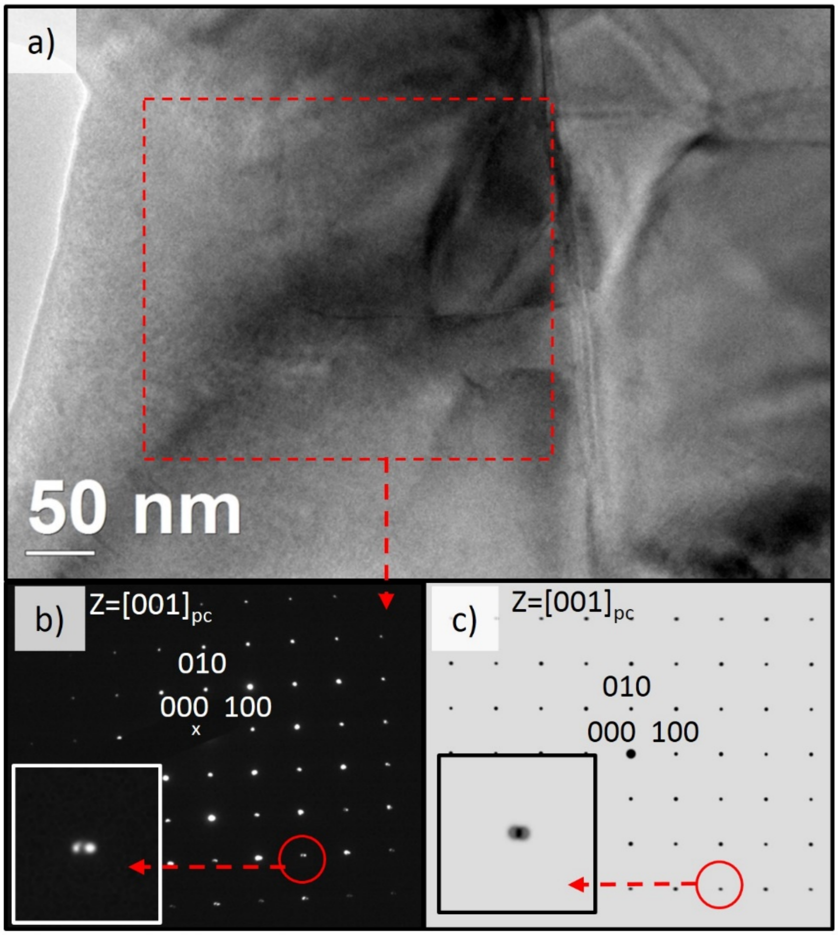

Supplementary Figure B1. Domain structure by TEM a) Bright-field (BF)-TEM image of the 15.5 mol% Sm grain with domains. b) Corresponding experimental SAED pattern in $[001]_{pc}$ zone axis where splitting of the marked (2 -3 0) reflection was observed in the $[100]_{pc}$ direction. c) Simulation of SAED spot splitting, where one domain in $[100]_{pc}$ and one in $[010]_{pc}$ zone axis (71° domains) were used.

In the manuscript attention is given to the domain size reduction as a function of the increasing Sm content and subsequent increase in Pbam phase composition (see manuscript text related to **Fig. 2**). We dismiss the grain size of the polycrystalline ceramics as a factor contributing to the changing domain size due to the consistent grain size in the range of 1–3 µm for all

materials. For further clarification, the average grain sizes determined for each composition are given here in **Supplementary Table B1**. The grain size was determined using scanning electron microscopy (SEM) imaging and a standard intercept method (ASTM Standard E112-13). The grain size values are comparable for each BSFO composition and did not exhibit a systematic reduction coinciding with the increasing Sm content. Thus, the reduction of domain size as a function of Sm content cannot be attributed to a variation in the grain size.

Supplementary Table B1. The average grain size of each sintered BSFO composition.

**Mol% Sm**	8	12	14	15.5	18
**Grain size (μm)**	2.0 ± 0.3	2.3 ± 0.5	2.3 ± 0.8	1.5 ± 0.3	1.8 ± 0.8

Supplementary material C

Electric-field induced phase transition

The present study reveals an electric-field induced phase transition between *Pbam* and *R*3*c* phases, during the application of a DC electric-field to the 15.5 mol% Sm composition (manuscript, **Fig. 4**). To further understand the behaviour of this transition during the switching strain evolution, we carried out bipolar strain-electric-field (S-E) investigations (manuscript, **Fig. 5**). During multiple repetitions of these strain investigations we also performed XRD, the results of which are presented in **Supplementary Fig. C1**. The data presented in this figure corresponds to that which is presented in the manuscript **Fig. 5**. XRD was performed on four separate samples of the composition closest to the morphotropic phase boundary (MPB) (15.5 mol% Sm), and XRD was conducted after each sample was subject to a pre-selected number of electric-field cycles (i.e., 1, 20 and 110 cycles) at the maximum amplitude (180 kV/cm) with frequency of 100 Hz.

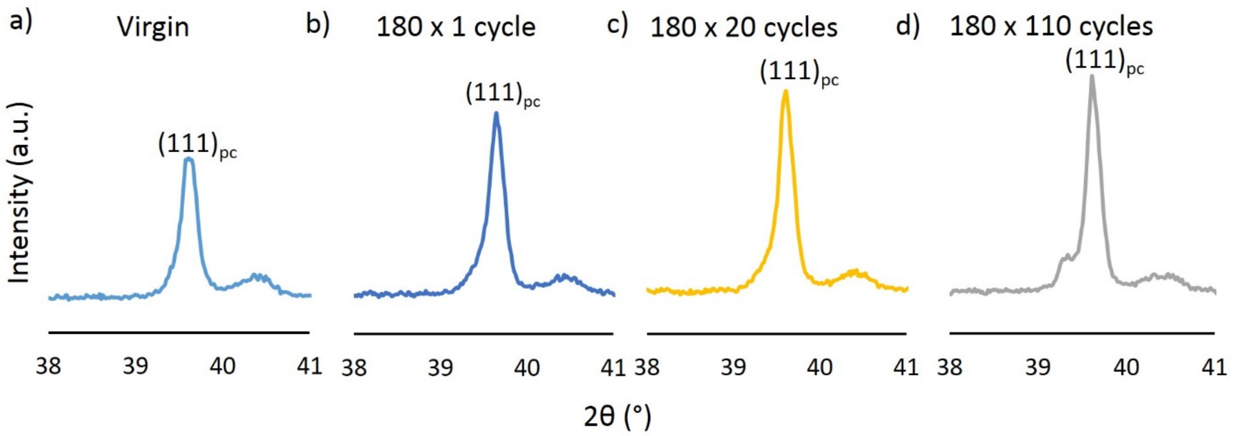

Supplementary Figure C1. XRD patterns of samples after specific number of cycles at 180 kV/cm and 100 Hz. a) zero cycles (virgin samples), b) 1 cycle, c) 20 cycles, d) 110 cycles.

The structural study reveals that the phase transition, as observed by *ex situ* XRD, is highly dependent on the specific parameters of the electric-field, i.e., frequency, magnitude and number of cycles. The DC electric-field experiments, where the maximum electric-field magnitude was 120 kV/cm and the field was applied once for a period of fifteen minutes, shows clear $(111)_{pc}$ peak splitting, indicative of the appearance of the R3c phase (manuscript, **Fig. 4 inset ii**). By comparison, the samples cycled under bipolar electric-fields at a maximum amplitude of 180 kV/cm and frequency of 100 Hz, only begin to exhibit $(111)_{pc}$ peak splitting after >~100 cycles (**Supplementary Fig. C1 d**). We note, that the structural data in both bipolar and DC electric-field experiments was collected *ex situ* (i.e., after removal of the electric-field), and thus, provides information on the residual phase transition, i.e., after relaxation.

A highly valuable method for observing the electric-field induced phase transition and its relaxation as a function of the electric-field parameters is *in situ* XRD during electric-field cycling using high-energy synchrotron radiations. We note that attempts have been made to perform this method however, the attempts have been unsuccessful thus far. This is largely due to the

limited current-voltage (power) supply, restrictions due to specific required sample dimensions and the fragile nature of the test apparatus.

In the absence of *in situ* structural data, useful information regarding the electric-field induced transition and its relaxation behavior can be attained from comparing the strain-electric-field (S-E) loops at low bipolar electric-fields (20 kV/cm) and different frequencies. We measured loops at 20 kV/cm amplitudes, in order to limit the strain influence from ferroelectric switching by being well below the ferroelectric coercive field (E_c) of ~130 kV/cm, as seen for BSFO (manuscript **Fig. 3**). It is worth noting that the E_c was also previously seen to be only weakly dependent on frequency [32].

At the electric-field amplitude of 20 kV/cm, large differences were seen as a function of frequency in the ″S″-like shape of the S-E loops, which arises as a result of the *Pbam*-to-*R*3*c* transition in BSFO (manuscript **Fig. 5a**). With the electric-field frequency of 100 Hz , the ″S″-like shape in the S-E loop was barely distinguishable above measurement noise, with a magnitude of 0.02% strain (**Supplementary Fig. C2**). When the frequency was significantly reduced to 0.01 Hz, the S-E loop exhibited peak-to-peak strains of 0.1% (**Supplementary Fig. C2**), i.e., on the level of ~160 nm of sample displacement. Thus the strain increased more than an order of magnitude for electric-fields with the same amplitude of 20 kV/cm and difference frequencies of 100 Hz and 0.01 Hz.

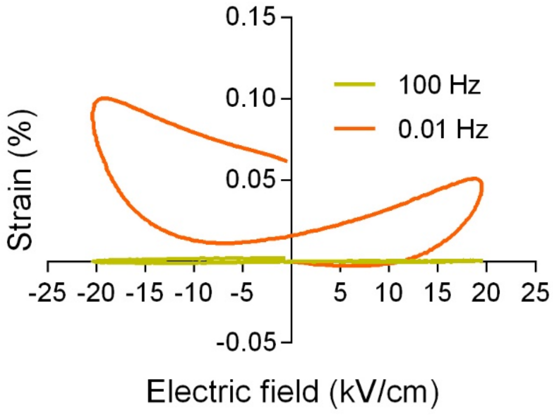

Supplementary Figure C2. Strain-electric-field loop of 15.5 mol% Sm materials cycled under an electric-field of 20 kV/cm. loops obtained with electric-field frequencies of 100 and 0.01 Hz are displayed.

This observation together with the dependence of residual *R*3*c* phase on the number of electric-field cycles (**Supplementary Fig. C1**), and the sluggish strain evolution of the material when subjected to cycling at electric-fields of 100 Hz (manuscript, **Fig. 5**), indicate the slow kinetics of the *Pbam*-to-*R*3*c* phase transition. We propose that this dependence is likely to arise due to a highly complex relationship between intergranular elastic strains and the phase transition, which is not entirely unexpected in a polycrystalline material when a maximum unit cell strain of ~0.8% (as discussed in the manuscript) is expected along the $[111]_{pc}$ direction as a result of the phase transition.

Supplementary material D

Calculation of maximum unit cell strain

The approximate unit cell strain discussed in the manuscript was calculated with **Supplementary Equation 1**, where S is the strain and d is the d spacing of the $\{111\}_{pc}$ lattice planes in the respective phases.

Supplementary Equation 1. $$S = \frac{d111_{R3c} - d111_{Pbam}}{d111_{R3c}}$$

The d spacing values were collected form the split $(111)_{pc}$ and $(1\ \text{-}1\ 1)_{pc}$ peaks of the XRD pattern after poling with a DC electric field, (seen in manuscript, **Fig. 4 inset ii**).

It is also worth briefly commenting on the intensity ratio of the $\{111\}_{pc}$ peaks after poling with a DC electric field (manuscript, **Fig. 4 inset ii**). Although a degree of peak overlapping of the $\{111\}_{pc}$ reflections is present between the *R*3*c* and *Pbam* phases, the intensity ratio of (111)/$(11\text{-}1)_{pc}$ reflections appears to be ~1:1, suggesting domain texture in the rhombohedral phase. Ideally for a sample with no domain texture in the rhombohedral phase this ratio should be 1:3. Thus, both the *Pbam*-to-*R*3*c* phase transition and domain switching of the resulting R3c phase take place during the application of the DC electric-field. This is consistent with the poled state of the samples which was confirmed by macroscopic measurement of the d_{33} (~25 pC/N).
